# Blue light promotes vascular reconnection, while red light boosts the physiological response and quality of grafted watermelon seedlings

**DOI:** 10.1038/s41598-021-01158-w

**Published:** 2021-11-05

**Authors:** Filippos Bantis, Emmanuel Panteris, Christodoulos Dangitsis, Esther Carrera, Athanasios Koukounaras

**Affiliations:** 1 Department of Horticulture, Aristotle University, 54124 Thessaloniki, Greece; 2 Department of Botany, School of Biology, Aristotle University, 54124 Thessaloniki, Greece; 3 Agris S.A., 59300 Kleidi, Imathia, Greece; 4 Institute of Molecular Biology and Plant Cell, Polytechnic University, 46022 Valencia, Spain

**Keywords:** Physiology, Plant sciences

## Abstract

The wound inflicted during grafting of watermelon seedlings requires rapid and sufficient vascular development which is affected by light quality. Our objective was to investigate the effect of light spectra emitted by light-emitting diodes (LEDs) during healing of grafted watermelon (*Citrullus lanatus*) seedlings on their vascular development, physiological and phytohormonal profile, and root architecture. Three LEDs emitting red (R), blue (B), and RB with 12% blue (12B) were tested in a healing chamber. During the first three days, the photosynthetic apparatus portrayed by PI_ABS_, φ_P0_, ψ_E0_, and ΔV_IP_ was less damaged and faster repaired in B-treated seedlings. B and 12B promoted vascular reconnection and root development (length, surface area and volume). This was the result of signaling cascade between phytohormones such as indole-3-acetic acid and others. After vascular reconnection the seedlings switched lights for 3 more days and the picture was reversed. Seedlings treated with B for the first 3 days and R for days 4 to 6 had better photosynthetic characteristics, root system development, morphological, shoot and root biomass, and quality (i.e. Dickson’s quality index) characteristics. We concluded that blue light is important during the first 3 days of healing, while the presence of red is necessary after vascular reconnection.

## Introduction

Vegetable grafting is a propagation technique that offers a number of benefits for crop production such as yield increase and tolerance to abiotic and biotic stresses^1^. Upon grafting, seedlings undergo a period of wound healing when histological and physiological modifications occur in vascular tissues adjacent to the grafting junction^2^. The stage of healing can be grouped into two stages; vascular reconnection and the subsequent growth boost. During healing, seedlings are carefully treated in environmentally controlled chambers with set conditions including temperature and relative humidity which must be close to 99% to minimize evapotranspiration. The latter is essential because of the absence of vascular bridges between the scion and rootstock segments which constitute the scion vulnerable and virtually incapable of development. Moreover, commercial nurseries usually have limited space available for healing of grafted seedlings, while demand is higher during specific seasons. Therefore, the potential for saving time is important for inceasing production quantity and reducing operational costs.

During healing light is necessary for normal seedling development. Plant development, morphology, and physiology are known to be regulated by light intensity, quality and duration^3,4^. Specifically, it is suggested that light quality affects the formation and activity of the photosynthetic apparatus^5^, as well as cell division^6^, adventitious rooting and root phototropism among other characteristics^7,8^, which are mainly modulated by phytohormones such as auxin, abscisic acid, jasmonic acid, and cytokinin among others. Phytohormones are signaling molecules found in plant tissues at very low concentrations and have a key role in regulating plants’ physiology, development and adaptation to environmental stimuli^9^. Auxin is considered as the main phytohormone responsible for the differentiation of vascular tissues, while other phytohormones interact with auxin to regulate this process^2^. Abscisic acid is known as the “stress phytohormone” and is biosynthesized in response to wound inflictions among other factors^10^, but its role in grafting is unclear yet.

The necessary energy and reducing power for all plant processes is generated through light interception, electron excitation and transfer via the electron transport chain where photosystem I (PS I) and PS II are the main structural components^11^. PS II is known to be regulated by light quality^5^, while the electron transport kinetics can enhance the understanding about spectral effects on the photosynthetic apparatus^12^. The induction curve of chlorophyll a fluorescence reveals a lot of information about plants’ photosynthetic apparatus. Specifically, OJIP transient provides an analysis of the electron transition between the PS II reaction centre, the cytochrome *b*_*6f.*_, the PS I reaction centre, and the end electron acceptors^11^. PI_ABS_, an index summarizing the activities of RC/ABS, φ_P0_, and ψ_E0_, is sensitive to different abiotic stress factors and is considered a successful tool to determine photosynthetic operation under varying environmental conditions (e.g. high temperature, salinity, nutrient deficiency etc.)^13^. Decrease of PI_ABS_ is associated with limitations in electron transport efficiency due to inhibited PS II activity^14^. Sood et al.^15^ observed severe PS I and PS II inhibition through proteins such as D1 (PS II reaction center), LHCPII (light-harvesting complex II), CP47 (PS II core antennae), and OEC33 (oxygen evolving complex), as well as partial inhibition of cytochrome *b*_*6f.*_ after exposure of wheat seedlings’ shoot–root transition zone to high irradiance (500 μmol m^−2^ s^−1^) of narrow-band red (670 nm) light. Light-emitting diodes (LEDs) are recently replacing traditionally used light sources such as fluorescent (FL) lamps because of a number of benefits including flexibility of spectral composition, high energy conversion efficiency, long operating lifetime, and low heat emission^16^.

Watermelon (*Citrullus lanatus*) is a popular crop with wide distribution from eastern Asia to the Mediterranean, and North America^1^. Due to successive cultivations in the same fields, soil-borne diseases and pests have become major issues during watermelon production, and since the use of methyl bromide is not permitted in the abovementioned regions, grafting onto resistant rootstocks is the best alternative for its propagation^17,18^. Moreover, a technique to increase grafting efficiency of cucurbit seedlings is to remove the root system which re-develops after a short period of time^19^. Therefore, the time needed for vascular reconnection and subsequent development of a newly formed vigorous root system is critical for the success and quality of grafted seedlings especially before exiting the healing chamber for acclimatization in a greenhouse. In Greece and other intensive production regions throughout Europe, grafted watermelon seedlings account for over 90% of all plants put into production, and high-quality seedlings are paramount.

Previous work has demonstrated the positive impact of certain red-blue LED wavelengths employed during the healing stage on the production of grafted watermelon seedlings^20^. At this stage we observed an interesting behavior involving the seedling quality under different light spectra before and after proper vascular formation, and we report it in the present study. However, no research articles have been published on the impact of light spectra on the intermediate stages (i.e. before and after vascular reconnection) of healing of grafted cucurbit seedlings including phytohormonal analysis. Moreover, information is scarce regarding light spectra effects on the vascular development, structural components of the photosynthetic apparatus, and root formation during that delicate stage. Therefore, the main objective of our study was to take a step forward and examine the influence of light spectra (narrow-band red or blue, and bichromatic red and blue) provided by LEDs, on the vascular reconnection, physiological (i.e. chlorophyll fluorescence) and phytohormonal profile, and root architecture of grafted watermelon seedlings during the first days after grafting (i.e. healing stage). In addition, we switched light treatments upon vascular reconnection (i.e. the third day after grafting) and studied the physiological status, root development, and general quality of the produced seedlings after their exposure to different light spectra before and after vascular reconnection.

## Materials and methods

### Plant material and grafting

The experiment was conducted in a commercial nursery (Agris S.A. in Kleidi, Imathia, Greece, http://www.agrishorticulture.com). “Celine F1”, a watermelon (*Citrullus lanatus*) hybrid was used as scion, while “TZ-148”, an interspecific squash (*Cucurbita maxima* × *C. moschata*) hybrid was used as rootstock. HM.Clause SA, Portes-Les-Valence, France, provided the seeds of both hybrids.

For the production of watermelon and interspecific squash seedlings to-be-grafted, seeds were sown in plug trays (171-cell and 128-cell, respectively), with a substrate comprised of peat, perlite and vermiculite, and they were stored in a dark chamber with temperature of 25 °C and high relative humidity (95–98%). Scion seeds were sown one day earlier than rootstock seeds in order to attain seedlings with similar stem diameter for successful grafting. After three days, the emerged seedlings were transferred in a greenhouse where temperature was controlled (21 °C minimum), and only watermelon was irradiated for 18 h with supplemental high-pressure sodium (HPS; MASTER GreenPower 600 W 400 V E40, Philips Lighting, Eindhoven, The Netherlands) lighting down to a minimum PPFD of 100 ± 10 μmol m^−2^ s^−1^.

During the “splice grafting” the whole root system was also removed, a beneficial technique for cucurbits as described by Lee and Oda^19^. Splice grafting is commonly practiced in Greek nurseries since it is 99% successful. The seedlings were grafted by experienced personnel of Agris S.A. and they were planted in 72–cell plug trays with a substrate comprised of peat, perlite and vermiculite.

### Healing stage, light conditions and treatment switch

Immediately upon grafting, three trays of grafted seedlings per light treatment were transferred in a healing chamber equipped with temperature (25 °C) and humidity (98%) controllers. Leaf dehydration is a crucial factor of grafting failure, thus high relative humidity is essential for its prevention. The seedlings remained in the healing chamber for six days.

Sole artificial lighting was applied by three LEDs with different radiation spectra, at 18 h photoperiod, and PPFD of 85 ± 5 μmol m^−2^ s^−1^. From unpublished observations we concluded that the optimum PPFD is 85 ± 5 μmol m^−2^ s^−1^ in order for the grafted watermelon seedlings to grow/heal without physiological disorders. The LEDs in our study did not have a dimmer connected, thus we placed them on carriage shelves at a distance of 30 cm from plant top which allowed us to irradiate the seedlings with the aforementioned PPFD. Specifically, LEDs emitted narrow-band red (R), narrow-band blue (B), and RB with 88% red and 12% blue (12B). 12B proved valuable for grafted watermelon seedlings’ quality in another study of our group^20^. Spectral parameters were obtained with a spectroradiometer (HD 30.1 spectroradiometer, DeltaOhm Srl, Padova, Italy) and are presented in Table 1.

**Table 1:** Spectral parameters of the three light treatments tested and abbreviated names of trays from each light treatment switching places with trays from the other two light treatments for days 4–6 after grafting.

Parameters	Light treatment			Light treatment at 6 days after grafting		
	R	B	12B	Abbreviation	Days 1–3	Days 4–6
UV %; 380–399 nm	0.08	0.09	0.05	R-R	R	R
Blue %; 400–499 nm	0.24	98.87	11.94	R-B	R	B
Green %; 500–599 nm	0.51	0.28	0.53	R-12B	R	12B
Red %; 600–699 nm	98.47	0.34	86.83	B-R	B	R
Far-red %; 700–780 nm	0.69	0.42	0.64	B-B	B	B
Blue peak wavelength (nm)	–	451	454	B-12B	B	12B
Red peak wavelength (nm)	661	–	660	12B-R	12B	R
R:B	–	–	7.27	12B-B	12B	B
PPS	0.89	0.51	0.89	12B-12B	12B	12B

The abovementioned light conditions applied for three days after grafting. At that point, two trays from each light treatment switched places with trays from the other two light treatments, while one tray remained in its initial light treatment leading to a total of 9 light treatments presented in Table 1. The trays remained in their final position for three more days (six days of healing in total) until exiting the healing chamber.

### Sampling and measurements

Relative chlorophyll content (measured with CCM-200 plus, Opti-Sciences, Hudson, NH, USA) and chlorophyll fluorescence measurements (measured with pocket plant efficiency analyzer, Hansatech, King’s Lynn, UK) were conducted in situ at several time intervals after a minimum of 20 min dark adaptation. Specifically, measurements were conducted after grafting at 6 h, 24 h, 48 h, 72 h, and 144 h on the sixth and final day of healing on 10 seedlings per time interval and light treatment. Regarding the induction curve of PS II chlorophyll fluorescence (OJIP transient), the following parameters are presented: PI_ABS_: performance index; φ_P0_: maximum quantum yield for primary photochemistry, ψ_E0_: probability that an electron moves further than Quinone; RC/ABS: Quinone reducing reaction centers per PSII antenna; and ΔV_IP_: relative fluorescence increase between the intersystem carriers and electron end acceptors of PS I^11^.

According to unpublished observations of our group, sufficient vascular reconnection in grafted watermelon seedlings takes place 3 days after grafting. On the third and sixth days after grafting, 10 seedlings per light treatment were sampled for morphological and biomass determinations. The determinations included parameters that are good quality indicators for grafted watermelon seedlings^21^. Specifically, stem length and diameter (i.e. rootstock diameter) were measured with a vernier caliper, while leaf area was measured with a leaf area meter (LI-3000C, LI-COR biosciences, Lincoln, USA). Shoot and root dry weight (oven-dried for 3 days at 72 °C) was determined with a precision scale. The above measurements were also used for the calculation of two useful quality indices, root-to-shoot (R/S) ratio and Dickson’s quality index (DQI) which was calculated as follows^22^.

$$DQI = \frac{{Seedling\;~total~\;dry~\;weight~\;\left( {\text{g}} \right)}}{{\frac{{Height\;~\left( {{\text{mm}}} \right)}}{{Stem~diameter\;~\left( {{\text{mm}}} \right)}}~ + ~\frac{{Shoot\;dry\;weight\;\left( {\text{g}} \right)}}{{Root~\;dry~\;weight~\;\left( {\text{g}} \right)}}~}}$$

Maximum tensile stress was determined as the tension required to separate the scion from the rootstock through the graft union. The rootstock was properly adjusted on the stable part (bottom) of a penetrometer using a vice, while the scion was viced on the moving part (top) of the penetrometer which was vertically moved until the graft union was broken.

In addition, root architecture parameters were determined in 6 seedlings per treatment at the third and sixth days after grafting. Specifically, root system parameters such as length, surface area (defined as the root area in contact with the soil), volume, and tip number were measured with WinRHIZO Pro software (Regent Instruments Inc., Québec, Canada) after scanning of meticulously rinsed roots.

Three and six days after grafting, 5 seedlings per treatment were sampled for microscopy analysis of the graft junction. In the sixth day, microscopy analysis was only performed on seedlings that remained for the whole period of healing in the same light treatment (i.e. R-R, B-B, and 12B-12B). The grafting junction was cut in slices including both scion and rootstock. The slices were fixed at room temperature into 4% paraformaldehyde in PBS, pH 7.0, for 60 min. After three washes with PBS, the slices were stained with propidium iodide (PI), 20 μg/mL in PBS, for 20 min. Afterwards, the slices were viewed with a confocal laser scanning microscope (CLSM, Zeiss LSM780, Carl Zeiss AG, Munich, Germany) with the appropriate filter set for PI, images were recorded with ZEN2011 software, while Adobe Photoshop was used for image processing.

### Phytohormone quantification

Three days after grafting, parts of the grafting junction from 3 seedlings per light treatment (only R-R, B-B, and 12B-12B in the sixth day) were divided into their original scion and rootstock segments. In previous experiments we observed that vascular reconnection is achieved after no more than 3 days, thus, molecular signals leading to phytohormonal production, accumulation, and transport are booming in the first three days after wounding/grafting. Therefore, we wanted to find out what phytohormonal modifications take place under each light quality in both segments (scion and rootstock), which are involved in the acceleration or deceleration of vasculature and root system formation. It must be noted that the plant material was very homogenous due to the controlled conditions during the seedlings’ production.

The segments were pulverized with liquid nitrogen and stored in deep freeze temperature (-80 °C) until phytohormonal quantifications. About 150 mg fresh weight per sample was suspended in methanol, water, acetic acid (80:19:1, v/v/v) containing internal standards and mixed by shaking during one hour at 4ºC. The extract was kept a -20ºCovernight and then centrifuged and the supernatant dried in a vacuum evaporator. The dry residue was dissolved in 1% acetic acid and passed through an Oasis HLB (reverse phase) column as described in Seo et al.^23^. For indole-3-acetic acid (IAA), abscisic acid, salicylic acid, and jasmonic acid quantification, the dried eluate was dissolved in 5% acetonitrile-1% acetic acid, and the hormones were separated using an autosampler and reverse phase UHPLC chromatography (2.6 µm Accucore RP-MS column, 100 mm length × 2.1 mmi.d.; ThermoFisher Scientific) with a 5 to 50% acetonitrile gradient containing 0.05% acetic acid, at 400 µL/min over 21 min. For cytokinins trans-zeatin, dihydrozeatin, and isopentenyladenine, the extracts were additionally passed through a Oasis MCX (cationic exchange) and eluted with 60% methanol- 5% NH_4_OH to obtain the basic fraction containing cytokinins. The final eluate was dried and dissolved in 5% acetonitrile-1% acetic acid and cytokinins were separated with a 5 to 50% acetonitrile gradient over 10 min. The hormones were analyzed with a Q-Exactive mass spectrometer (Orbitrap detector; ThermoFisher Scientific) by targeted Selected Ion Monitoring (SIM). The concentrations of hormones in the extracts were determined using embedded calibration curves and the Xcalibur 4.0 and TraceFinder 4.1 SP1 programs. The internal standards for quantification of each of the different plant hormones were the deuterium-labelled hormones, except for jasmonic acid, for which the compound dhJA was used.

### Statistical analysis

The experiment was performed twice reaching similar conclusions. Statistical analysis was conducted using IBM SPSS software (SPSS 23.0, IBM Corp.). Specifically, in all cases analysis of variation (ANOVA) was employed for data analysis at significance level *p* = 0.05, while mean comparisons were conducted using Tukey post-hoc test at significance level of *p* = 0.05.

### Ethics declaration

All the protocols adhered to relevant plant ethics guidelines.

## Results

**Figure 1:**
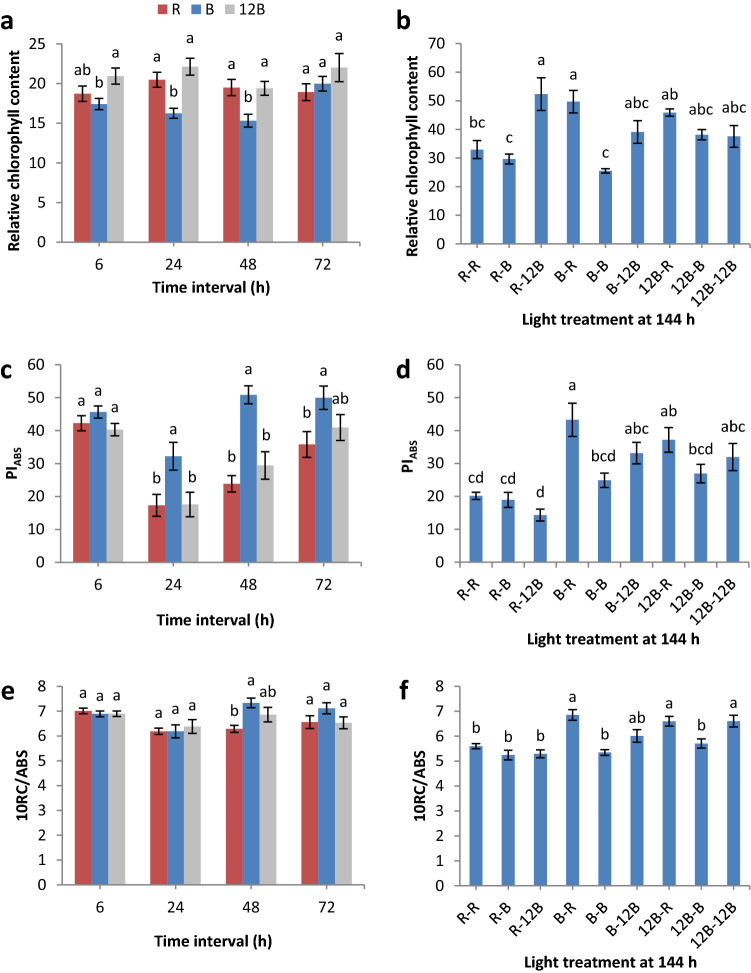
Relative chlorophyll content (**a**,**b**), performance index on absorption basis (PI_ABS_) (**c**,**d**), and density of active reaction centres (10RC/ABS) (**e**,**f**) of grafted watermelon seedlings at four time intervals under three light treatments (**a**,**c**,**e**) and at day 6 (144 h) under nine light treatments (**b**,**d**,**f**) in the healing chamber. Bars (± SE) within a time interval followed by different letters are significantly different (a < 0.05). Mean values derived from n = 10 seedlings.

**Figure 2:**
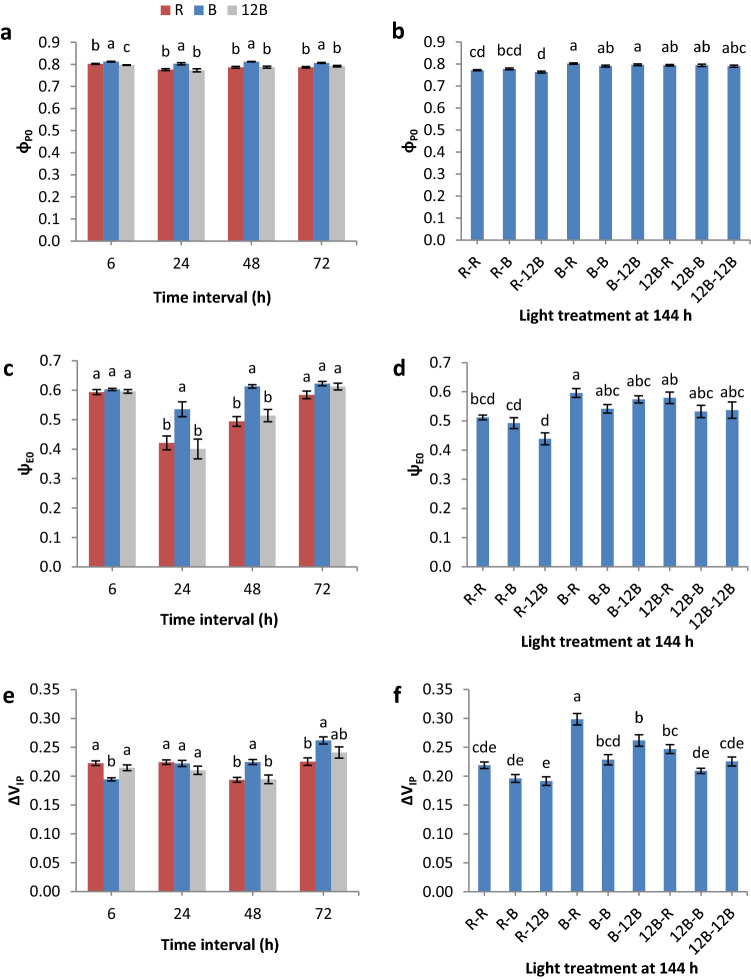
Quantum efficiency of reduction of Q_A_ (φ_P0_) (**a**,**b**), probability of electron transport beyond Q_A–_ (Ψ_E0_) (**c**,**d**), and relative fluorescence increase between I- and P-step (ΔV_IP_) (**e**,**f**) of grafted watermelon seedlings at four time intervals under three light treatments (**a**,**c**,**e**) and at day 6 (144 h) under nine light treatments (**b**,**d**,**f**) in the healing chamber. Bars (± SE) within a time interval followed by different letters are significantly different (a < 0.05). Mean values derived from n = 10 seedlings.

The plants’ photosynthetic apparatus can efficiently be studied by examining the induction curve of chlorophyll a fluorescence. Seedlings treated with B and 12B during days 1 to 3, and seedlings treated with R during days 4 to 6 after grafting responded better regarding their photosynthetic potential. Specifically, we found that relative chlorophyll content was significantly lower under B compared to 12B at 6, 24, and 48 h after grafting, and compared to R at 24 and 48 h after grafting (Fig. 1a), while R-R, R-B, and B-B had significantly lower values compared to R-12B and B-R at 144 h after grafting (Fig. 1b). PI_ABS_ was significantly greater under B compared to R at 24, 48, and 72 h after grafting, and compared to 12B at 24 and 48 h after grafting (Fig. 1c), while B-R had significantly greater values compared to R-R, R-B, R-12B, B-B, and 12B-B at 144 h after grafting (Fig. 1d). Regarding 10RC/ABS, we observed that B led to greater values compared to R at 48 h (Fig. 1e), while B-R, 12B-R, and 12B-12B led to greater values compared to R-R, R-B, R-12B, B-B, and 12B-B at 144 h after grafting (Fig. 1f). φ_P0_ was significantly greater under B compared to both 12B and R at 6, 24, 48, and 72 h after grafting (Fig. 2a), while B-R and B-12B had significantly greater values compared to R-R, R-B, and R-12B at 144 h after grafting (Fig. 2b). Quite similarly, ψ_E0_ revealed greater values under B compared to 12B and R at 24 and 48 h after grafting (Fig. 2c), while B-R revealed greater values compared to R-R, R-B, and R-12B at 144 h after grafting (Fig. 2d). Regarding ΔV_IP_, B induced the lowest values at 6 h but the greatest values at 48 and 72 h (Fig. 2e), while B-R induced the greatest values compared to the rest of the light treatments at 144 h after grafting (Fig. 2f).

**Figure 3:**
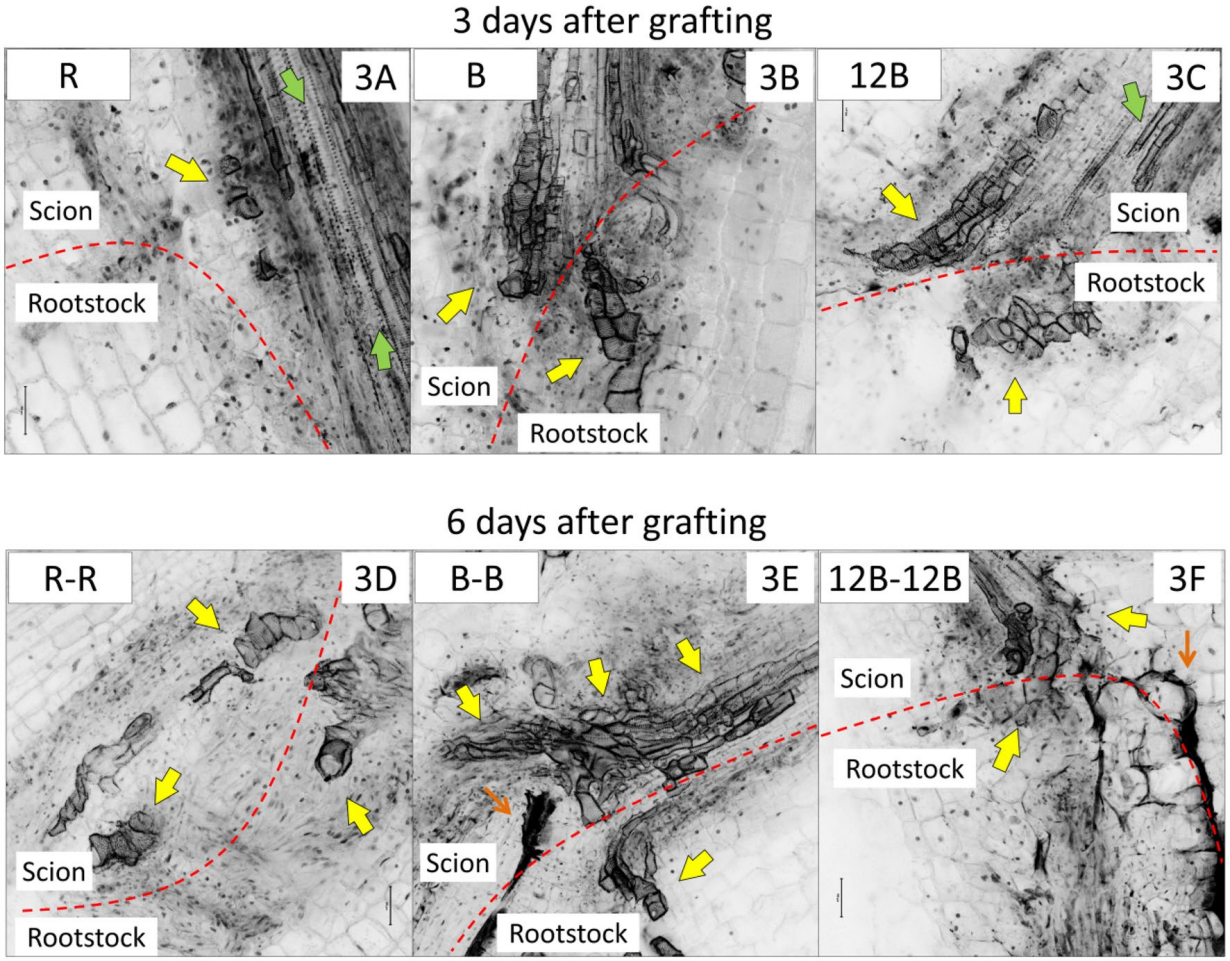
Projections of CLSM sections, depicting the histological organization at the grafting junction of grafted watermelon seedlings after three (upper line) or six (bottom line) days in the healing chamber under three light treatments. In all the CLSM figures, fluorescence intensity has been inverted (negative image) for better visualization. Red dashed lines indicate the grafting junction line. Yellow arrows indicate newly formed vessels which derived from redifferentiated parenchymatic cells. Green arrows indicate initial xylem vessels. Thin orange arrows indicate wound cork that was formed at some sites of the junction.

A rapidly reconstructed vasculature is essential for fast seedling growth during healing. Figure 3 depicts the anatomical structure at the grafting junction 3 and 6 days after grafting. On day 6 after grafting, the CLSM observations were similar in all light treatments, thus we opted to show the treatments where seedlings remained for the whole healing period (i.e. R-R, B-B, and 12B-12B). Similarly, supplementary Figure S1 depicts another batch of CLSM observations for each treatment. After 3 days, it is clearly shown that B-treated seedlings had higher vascular tissue regeneration, connecting through by-pass the severed vascular bundles. As typical, the initially formed vessels (during primary growth) of the xylem are highly elongated and narrow (see indicative green arrows in Figs. 3 and S1). Since the newly formed vessels (see indicative yellow arrows in Figs. 3 and S1) derive from redifferentiated parenchymatic cells, they are short, irregularly shaped, also with irregular secondary wall thickenings. A schematic depiction of histological organization at the grafting junction is shown in supplementary Figure S2. After 6 days, vascular reconnection was relatively progressed under all light treatments and no particular differences were observed.

**Figure 4:**
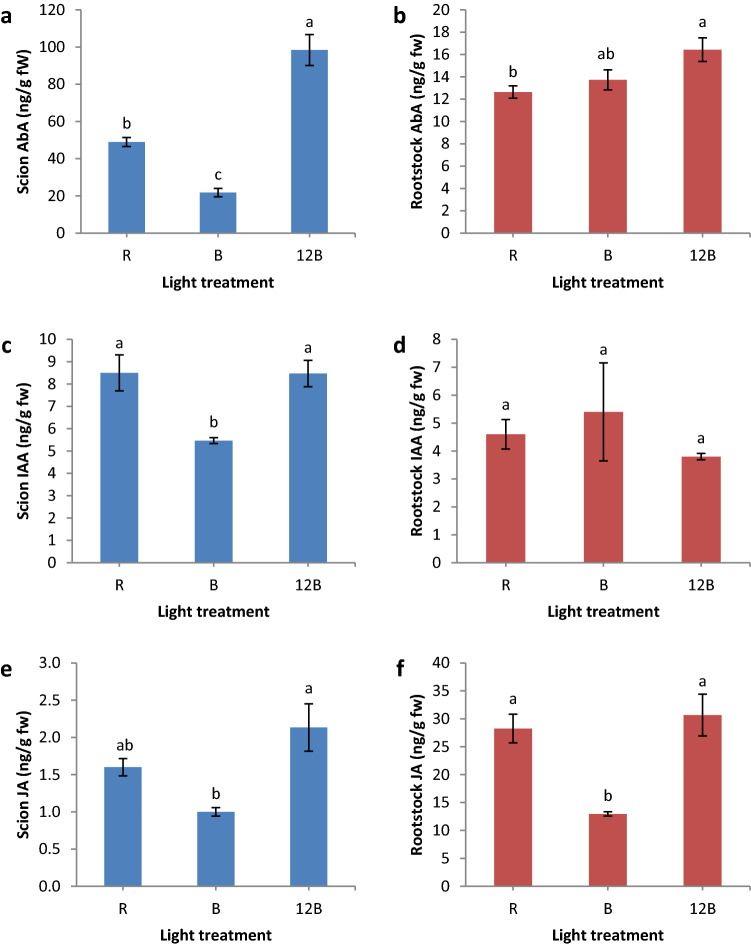
Abscisic acid (AbA) (**a**,**b**), indole-3-acetic acid (IAA) (**c**,**d**), and jasmonic acid (JA) (**e**,**f**) of scion (**a**,**c**,**e**) and rootstock (**b**,**d**,**f**) segments of grafted watermelon seedlings under three light treatments in the healing chamber at three days after grafting. Bars (± SE) followed by different letters are significantly different (a < 0.05). Mean values derived from n = 3 seedlings.

**Figure 5:**
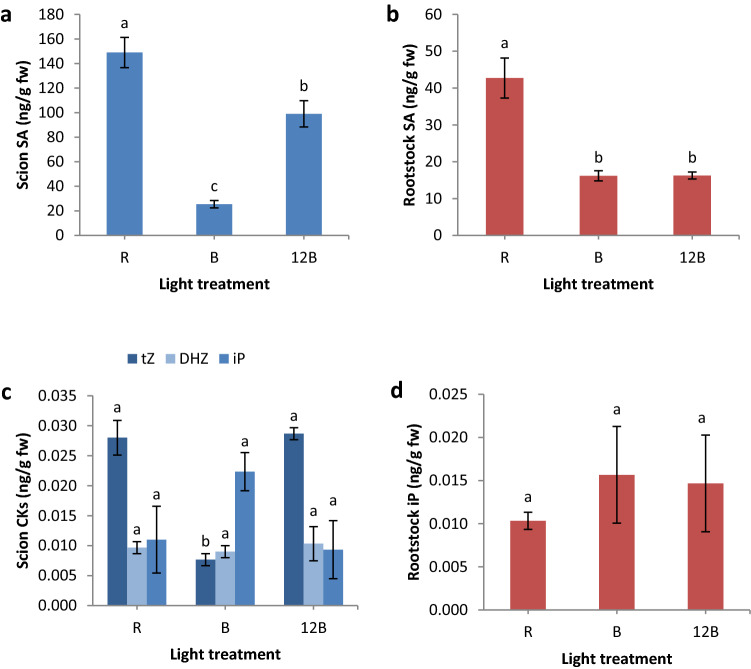
Salicylic acid (SA) (**a**,**b**) and cytokinins (CKs) trans-zeatin (tZ), dihydrozeatin (DHZ) (**c**), and isopentenyladenine (iP) (**c**,**d**) of scion (**a**,**c**) and rootstock (**b**,**d**) segments of grafted watermelon seedlings under three light treatments in the healing chamber at three days after grafting. Bars (± SE) followed by different letters are significantly different (a < 0.05). Mean values derived from n = 3 seedlings.

Vasculature formation is regulated by several phytohormones, thus, we conducted separate phytohormonal analysis for scion and rootstock segments at 3 days after grafting and we present the results in Figs. 4 and 5. Briefly, the scion segment developed under B formed significantly less abscisic acid (-351 and -125%, respectively), IAA (both -55%), salicylic acid (-289 and -486%, respectively), and trans-zeatin (-200 and -167%, respectively) compared to 12B and R, and significantly less jasmonic acid compared to 12B (-113%), while dihydrozeatin and isopentenyladenine were not significantly affected by the light treatments. Regarding the rootstock segment, we found that abscisic acid was greater under 12B compared to R (+ 30%), jasmonic acid was greater under 12B and R compared to B (+ 137 and + 118%, respectively), and salicylic acid was greater under R compared to B and 12B (+ 264 and + 263%, respectively), while IAA and isopentenyladenine were not significantly affected. We also quantified gibberellins GA_1_ and GA_4_, but the samples showed highly irregular variability. Specifically, scion GA_1_ was 445.39 ± 444.62 (standard error) in R, 63.19 ± 39.74 in B, and 1.39 ± 0.09 in 12B, while rootstock GA_1_ was 109.36 ± 99.72 in R, 321.27 ± 314.58 in B, and 13.57 ± 0.64 in 12B. Scion GA_4_ was 407.83 ± 404.01 in R, 23.13 ± 12.35 in B, and 1.00 ± 0.39 in 12B, while rootstock GA_4_ was 30.54 ± 23.10 in R, 145.48 ± 144.52 in B, and 0.19 ± 0.03 in 12B.

The analysis of root architecture provides valuable information involving the root system development which was generally enhanced by B light in the first 3 days, while R and 12B promoted its growth in the following days (4 to 6). Table 2 depicts root architectural parameters at 3 and 6 days after grafting. Three days after grafting, B-treated seedlings enhanced root parameters compared to 12B and R, showing longer roots (+ 108 and + 90%, respectively), greater surface area (+ 68 and + 70%, respectively), and greater root volume (+ 50% compared to R), while tip number was not significantly affected. Six days after grafting, we found that R-B treated seedlings had lower length compared to B-R, B-12B, and 12B-R (the latter were + 105, + 105, and + 126%, respectively, compared to R-B), lower root volume compared to 12B-R and 12B-12B (the latter were + 85 and + 75%, respectively, compared to R-B), and smaller surface area compared to B-R, B-12B, 12B-R, and 12B-12B (the latter were + 77, + 85, 106, and + 81%, respectively, compared to R-B), while B-B also had smaller surface area compared to 12B-R (+ 60% compared to R-B). At the same time interval (6 days after grafting) and regardless of the light spectra provided at days 1–3, we observed that seedlings treated with B during days 4–6 had significantly lower root length, surface area, and root volume compared to R and 12B (data not shown).

**Table 2:** Root architecture parameters of grafted watermelon seedlings analyzed with WinRHIZO root-scanning system after three or six days in the healing chamber under three or nine light treatments, respectively.

Light treatments	Length (cm)	Surface area (cm^2^)	Volume (cm^3^)	Tip No
Days 1–3 after grafting				
R	28.7 ± 5.7 b	8.0 ± 1.4 b	0.20 ± 0.03 b	155.4 ± 25.5 a
B	48.9 ± 8.0 a	13.7 ± 1.8 a	0.31 ± 0.03 a	256.5 ± 81.0 a
12B	23.5 ± 3.1 b	8.1 ± 0.1 b	0.22 ± 0.02 ab	82.7 ± 9.3 a
Days 4–6 after grafting				
R-R	156.9 ± 23.4 ab	26.2 ± 3.7 abc	0.35 ± 0.04 ab	1376.0 ± 298.8 a
R-B	102.1 ± 5.4 b	17.3 ± 0.7 c	0.24 ± 0.03 b	774.3 ± 164.4 a
R-12B	155.3 ± 10.6 ab	26.8 ± 1.1 abc	0.37 ± 0.03 ab	1296.3 ± 246.0 a
B-R	209.7 ± 33.5 a	30.5 ± 2.9 ab	0.36 ± 0.02 ab	1618.4 ± 480.4 a
B-B	148.2 ± 26.0 ab	22.3 ± 3.4 bc	0.27 ± 0.04 ab	1025.3 ± 186.0 a
B-12B	209.3 ± 21.8 a	31.9 ± 2.3 ab	0.39 ± 0.02 ab	1754.0 ± 313.0 a
12B-R	230.2 ± 20.6 a	35.6 ± 4.3 a	0.44 ± 0.07 a	1734.3 ± 104.2 a
12B-B	155.8 ± 8.5 ab	24.1 ± 2.1 abc	0.30 ± 0.04 ab	1401.8 ± 131.1 a
12B-12B	188.0 ± 10.6 ab	31.3 ± 1.4 ab	0.42 ± 0.02 a	1675.0 ± 212.6 a

Mean values (± SE) within a column followed by different letters indicate significant differences (a < 0.05). Mean values derived from n = 6 scanned roots.

The examination of morphological and biomass characteristics is essential in order to evaluate the practicality of the produced seedlings, whose overall quality was suppressed when treated with B during days 4 to 6 after grafting. Regarding the morphological and biomass parameters, and quality indices obtained 3 days after grafting, we found no differences in stem diameter, leaf area, maximum tensile stress, and shoot dry weight. However, we found that stem length was greater under 12B compared to R (+ 15%), while B had greater root dry weight (+ 37%) and DQI (+ 27%) compared to 12B, and greater R/S ratio compared to 12B and R (+ 45 and + 36%, respectively) (Table 3). Six days after grafting, seedlings treated with R-12B and B-12B had greater stem diameter compared to 12B-B (both + 111%), seedlings treated with 12B-12B had greater leaf area compared to R-B, B-B, and 12B-B (+ 64, + 63, and + 53%, respectively), and seedlings treated with 12B-12B had greater maximum tensile stress compared to R-R, R-B, and B-B (+ 107, + 59, and + 59%, respectively), while stem length was not significantly affected. Moreover, we observed that shoot dry weight was greater under B-12B, 12B-R, and 12B-12B compared to R-B (+ 29, + 33, and + 26%, respectively), root dry weight was greater under 12B-R compared to R-B and B-B (+ 82 and + 72%, respectively), R/S ratio was greater under B-R compared to B-B (+ 60%), and DQI was greater under 12B-12B compared to R-B (+ 68%) (Table 3). At 6 days after grafting and regardless of the light treatment at days 1–3, we found that seedlings treated with B during days 4–6 had significantly lower values in leaf area, stem diameter, shoot and root dry weight, R/S ratio, and DQI compared to R and 12B, while 12B led to greater maximum tensile stress compared to R and B (data not shown).

**Table 3:** Morphological parameters, biomass production, and quality indices of grafted watermelon seedlings after three or six days in the healing chamber under three or nine light treatments, respectively.

Light treatments	Stem length (mm)	Stem diameter (mm)	Leaf area (cm^2^)	Maximum tensile stress (kg)	Shoot dry weight (g)	Root dry weight (g)	R/S ratio	DQI × 1000
Days 1–3 after grafting								
R	35.85 ± 1.47 b	3.82 ± 0.06 a	5.52 ± 0.52 a	0.095 ± 0.020 a	0.159 ± 0.005 a	0.023 ± 0.001 b	0.144 ± 0.011 b	11.54 ± 0.57 ab
B	37.73 ± 1.57 ab	3.92 ± 0.06 a	6.17 ± 0.34 a	0.135 ± 0.027 a	0.162 ± 0.003 a	0.031 ± 0.003 a	0.195 ± 0.015 a	13.31 ± 0.77 a
12B	41.23 ± 1.28 a	3.77 ± 0.09 a	5.05 ± 0.52 a	0.158 ± 0.017 a	0.168 ± 0.003 a	0.023 ± 0.002 b	0.135 ± 0.013 b	10.49 ± 0.95 b
Days 4–6 after grafting								
R-R	34.44 ± 2.79 a	4.18 ± 0.08 ab	15.20 ± 2.56 abcd	0.300 ± 0.047 c	0.149 ± 0.007 ab	0.031 ± 0.002 ab	0.214 ± 0.007 ab	12.31 ± 1.33 ab
R-B	34.44 ± 0.75 a	4.00 ± 0.09 ab	12.42 ± 1.41 d	0.390 ± 0.033 bc	0.127 ± 0.006 b	0.022 ± 0.004 b	0.171 ± 0.024 ab	10.08 ± 1.19 b
R-12B	39.38 ± 1.75 a	4.33 ± 0.08 a	16.80 ± 1.61 abcd	0.440 ± 0.024 abc	0.143 ± 0.004 ab	0.031 ± 0.003 ab	0.211 ± 0.014 ab	13.26 ± 1.14 ab
B-R	35.62 ± 1.74 a	4.14 ± 0.09 ab	19.45 ± 0.67 ab	0.470 ± 0.037 abc	0.153 ± 0.005 ab	0.036 ± 0.002 ab	0.246 ± 0.022 a	14.49 ± 0.76 ab
B-B	35.91 ± 2.58 a	3.98 ± 0.09 ab	12.45 ± 1.17 cd	0.390 ± 0.043 bc	0.151 ± 0.010 ab	0.023 ± 0.002 b	0.154 ± 0.020 b	11.04 ± 1.07 ab
B-12B	33.50 ± 1.62 a	3.98 ± 0.08 a	19.32 ± 1.29 abc	0.510 ± 0.040 ab	0.164 ± 0.007 a	0.035 ± 0.004 ab	0.215 ± 0.018 ab	15.74 ± 1.90 ab
12B-R	37.65 ± 1.03 a	4.35 ± 0.12 ab	20.08 ± 1.53 ab	0.480 ± 0.037 abc	0.169 ± 0.009 a	0.040 ± 0.004 a	0.217 ± 0.013 ab	16.32 ± 1.68 ab
12B-B	35.65 ± 1.07 a	3.90 ± 0.11 b	13.26 ± 1.02 bcd	0.490 ± 0.029 abc	0.146 ± 0.006 ab	0.027 ± 0.002 ab	0.182 ± 0.023 ab	11.74 ± 0.72 ab
12B-12B	34.18 ± 2.87 a	4.18 ± 0.07 ab	20.32 ± 1.64 a	0.620 ± 0.068 a	0.160 ± 0.005 a	0.036 ± 0.004 ab	0.223 ± 0.020 ab	16.92 ± 2.18 a

*R/S* dry root-to-shoot ratio; *DQI* Dickson quality index. Mean values (± SE) within a column followed by different letters are significantly different (a < 0.05). Mean values derived from n = 10 seedlings.

## Discussion

Vegetable grafting imposes significant stress on seedlings and thus wound healing is critical for their survival and proper development. Apart from important environmental conditions such as temperature and relative humidity which are well-established, light is a highly influential factor during healing. By controlling light wavelength in particular we have the potential to fine-tune morphological and physiological responses and regulate seedling quality. Incident light is captured and utilized by plant pigments and photoreceptors that trigger a signaling cascade. Red and blue lights are the main contributors to the above mentioned responses and are perceived by pigments such as chlorophylls and carotenoids, and photoreceptors such as phytochromes, cryptochromes, phototropins and members of Zeitlupe family^24–27^.

In general, PI_ABS_, RC/ABS, φ_P0_, and ψ_E0_ showed a similar trend where the lowest values were observed at 24 h followed by a steady increase up to 72 h after grafting. It is clear that 6 h were insufficient for the different light spectra to cause substantial damage to the photosynthetic apparatus. Seedlings treated with B (mainly) and 12B (secondarily) performed considerably better than seedlings treated with R for the first 3 days after grafting. Moreover at 144 h after grafting, in the abovementioned parameters including ΔV_IP_, the lowest values were mainly observed in seedlings treated with R for the first 3 days after grafting (i.e. R-R, R-B, and R-12B), and secondarily in seedlings treated with B for days 4 to 6 after grafting (i.e. R-B, B-B, and 12B-B). On the contrary, as a general rule seedlings treated with B or 12B for the first 3 days after grafting and R for days 4 to 6 after grafting (i.e. B-R and 12B-R) reached higher values.

In the absence of blue light, red light presumably damages the PS II antenna, while blue light may restore it within 3 to 5 days as reported by Schmid et al.^28^ for *Acetabularia mediterranea* algae. In this context, red light limits the biosynthesis of core antenna proteins such as CP43 and CP47 subsequently leading to uncoupling between PS II antennas and reaction centres. In Arabidopsis, only 5 μmol m^−2^ s^−1^ of blue light (470 nm) acting through cryptochromes have been found to induce the expression of *psbD*, the gene encoding D2 protein which is a major structural component of PS II^29^. In our case, grafted watermelon seedlings treated with R for the first 3 days after grafting (i.e. R-R, R-B, and R-12B) probably encountered insufficiently coupled PS II core antennas and reaction centres, while this effect was not reversed upon providing blue light for days 4 to 6 after grafting. This observation was evident in all tested chlorophyll fluorescence parameters. Moreover, the significant decrease of RC/ABS in R-treated seedlings may signify inactivated reaction centres and Q_A_ reducing centres^30^.

Hosseini et al.^12^ examined chlorophyll a fluorescence of green and purple basil and found a clear suppression of PI_ABS_, RC/ABS, φ_P0_, and ψ_E0_ in R-treated plants compared to blue, white, and red-blue combinations which coincide with the results of our study. Seedlings of another cucurbit, cucumber, were exposed to low light intensity (50 μmol m^−2^ s^−1^) and exhibited significantly greater φ_P0_ and PI_ABS_ values under blue or white compared to red, while chlorophyll content was the least under blue^31^. The authors concluded that narrow-band blue was critical for the stability of the photosynthetic function while low chlorophyll content was beneficial for the photosynthetic activity maintenance suggesting that blue light can maintain photosynthesis under low light for a long duration. Baker^32^ reported that φ_P0_ values below 0.80 are associated with down-regulation of PS II activity due to stressful conditions. In a study with cucumber, seedlings exposed to blue light also exhibited lower chlorophyll content values compared to red, but higher values were found in red-blue combinations with increasing blue light^33^. The authors pointed out a substance transport problem within a red-light exposed leaf which had obvious heterogenous φ_P0_ distribution between the veins and the rest of the blade. Our ψ_E0_ results show that seedlings treated with R for the first 3 days after grafting were less efficient in conveying electrons into the electron transport chain and beyond Q_A_ proving inferior in regulating the electron level in the reaction centres^11^.

Vascular reconnection is necessary to be quick in order for seedlings to boost their development. During the first three days of grafted watermelon healing, confocal analysis exhibited B light’s positive effect on the formation of vasculature bridging the scion and rootstock segments. At this time interval, a signaling cascade involving phytohormone biosynthesis and transportation triggered the vasculature reformation at a significantly more rapid pace under the influence of B light compared to R light and even 12B which demonstrates the importance of a minimum threshold of blue light. The improved vascular formation in 12B shows that blue light is essential in relatively low amounts (e.g. about 10 μmol m^−2^ s^−1^ of 12B) and not only in large amounts such as B (about 85 μmol m^−2^ s^−1^). By determining the maximum tensile stress in the grafting junction we attempted to quantify the scion-rootstock connection and vascular bundle formation as affected by light quality. After 3 days of light irradiance in the healing chamber, increased maximum tensile stress was found under 12B followed by B and R but values were not significantly different. Similar trend was observed by Lee et al.^34^ who reported increased maximum tensile stress in grafted tomato seedlings treated with blue or white-red-blue (W1R2B1) compared to red after 4 days of grafting. After the second set of lighting (days 4 to 6 after grafting) when sufficient vascular connection was already achieved under all light treatments, maximum tensile stress was the least in seedlings treated with R during days 1 to 3 and seedlings treated with B during days 4 to 6 after grafting (i.e. R-R, R-B, and B-B).

Almost every phytochrome participates in the regulation of vascular tissues’ development and differentiation^2^. Even though the vascular reconnection was quicker in B-treated seedlings, phytohormonal analysis revealed reduced abscisic acid, IAA, jasmonic acid, salicylic acid, and trans-zeatin contents under B light compared to R and 12B. *Arabidopsis* grafts exhibited a symmetric auxin response on the top and below part of the grafting junction from 12 h after grafting, consistently with the auxin-inducing genes *DR5, IAA5, and ANAC071* within the first three days after grafting^35^. The same study states that proteins such as ALF4 or AXR1 possibly promote auxin response and vascular regeneration on the rootstock segment, an important feature in cases of inefficient transport such as the first days after grafting. Sauer et al.^36^ reported that auxin acts as polarizing signal allowing cells and tissues to be oriented correctly and relative to existing body structures. Balla et al.^37^ found that auxin efflux carrier PIN1 polarization induces auxin transport channels and vascular strands. In addition, it was found that RNA signal movement is faster from shoot to root compared to the opposite direction^38^. Meng et al.^39^ found enhanced lateral root development in tobacco seedlings under the influence of red light which promoted endogenous IAA concentration in roots. The authors concluded that red light induced polar transport of auxin from the leaves to the roots through the expression of *PIN3* genes, while blue light caused an underexpression of *PIN1*, *PIN3*, and *PIN4* genes involved in auxin transport regulation. In a study with tomato, blue light was reported to promote the formation of gibberellin GA_19_ which is a precursor of an important plant gibberellin, GA_1_^40^. The biosynthesis and accumulation of gibberellins are reportedly activated by auxins, while gibberellins participate in auxin transport through modulation of PIN proteins’ turnover^2,41,42^. Therefore, it is evident that gibberellins have a positive role in cell expansion and wound healing, while auxin promotes cell proliferation and vascular formation in grafted seedlings^43,44^. In our study gibberellins GA_1_ and GA_4_ were also quantified but variability was highly irregular.

Leaves and petioles of *Brassica napus* seedlings contained more IAA and less abscisic acid under light with increased red/far-red ratio^45^. In a review compiled by Nanda and Melnyk^2^ it is stated that abscisic acid signaling converges with vascular formation on the homeodomain-leucine zipper-type transcription factor, ARABIDOPSIS THALIANA HOMEOBOX 7, a gene expressed in tissues such as differentiating xylem. Regarding cytokinin quantifications, B light induced lower trans-zeatin content in the scion segment compared to R and 12B. In *Arabidopsis*, wounding promotes cytokinin-related responses involving callus formation through the transcription factor WOUND INDUCED DEDIFFERENTIATION 1 which activates cytokinin signaling^2^. Cytokinins act as auxin antagonists and impose negative regulation of xylem differentiation, as reported by Yokoyama et al.^46^. This statement possibly explains the greater and quicker vascular development in seedlings grown under B light, which allowed auxin to transport from the scion to the rootstock segment and regulate the root development. Jasmonic acid accumulates as a response to wounding and its biosynthesis is suppressed by the auxin response factors ARF6 and ARF8^47^. In our study, jasmonic acid reached significantly higher values in the rootstock segment, but B-treated seedlings revealed lower values. In *Arabidopsis* plants, the jasmonic acid precursor cis-12-oxo-phytodienoic acid reportedly translocated from wounded tissues to the root system where it participated in the regulation of jasmonic acid responses regarding the hormone’s signaling and its conversion into bioactive hormone jasmonoyl-L-isoleucine^48^, showing its direct involvement with the root system development.

Root scanning is a useful method of visually depicting and analyzing the root architecture as affected by different factors. Pang et al.^49^ suggested that WinRHIZO root-scanning system provides a more detailed morphological and architectural analysis of the root system compared to other methods. In our case, the root system development variable responded during the first (days 1 to 3) and last (days 4 to 6) days of healing. At the first 3 crucial days after grafting, it is evident that B light promoted the rapid formation of lateral roots while R decelerated root growth. As discussed above, B light triggered the rapid vasculature formation leading to quick lateral root formation. Under B, the root system size suggests that vascular reconnection started even earlier than the third day after grafting. However, at 6 days after grafting, seedlings from all light treatments developed several vascular bridges. Seedlings treated with red-containing light spectra (R or 12B) during days 4 to 6 after grafting developed a larger root system compared to B-treated seedlings. At this time point, R and 12B imposed a significant effect leading to a faster root development through the formation of an expanded lateral root system. The differences in surface area are mainly associated with thinner diameter classes (diameter between 0.5 and 1.5 mm) where similar differences were exhibited (data not shown). Phytochrome (photoreceptor of red and far-red lights) regulates the formation of lateral roots through the distribution of auxin which is transferred from shoot to root^50^. As previously described, IAA biosynthesis in the scion was enhanced by red light and was possibly transported (auxin transportation was not quantified in this manuscript) in the rootstock to regulate root system development. Similarly to our findings, artichoke seedlings developed longer roots when treated with red LED compared to white or blue LEDs^51^.

Although critical for examining a seedling’s quality, morphological characteristics are affected at a slower pace by the different light spectra compared to physiological and phytohormonal characteristics. Therefore, 3 days of light irradiance in the healing chamber did not impose significant effects on most above-ground parameters. In a recent publication, leaf area and stem diameter were characterized as important indicators for the quality of grafted watermelon seedlings^21^. In the current study, the presence of red light during days 4 to 6 after grafting promoted leaf expansion, reaffirming previous results of our group^20^. Moreover, it is evident that seedlings treated with B for days 4 to 6 after grafting limited the development of stem diameter and leaf area even in seedlings exposed to B during the first 3 days (i.e. leaf area of B-B). This is not surprising since red light responses include leaf area regulation through signaling by phytochrome photoreceptors^24,33^, while blue light inhibits leaf expansion through cryptochrome photoreceptors^27^. However, the fact that B significantly enhanced vascular development and root system formation in the first three days, but R surpassed it after only three days is surprising and is reflected in these two important quality indices. In another study with tomato seedlings, narrow-band red light and R3/B1 combinations enhanced the stem diameter compared to white and blue LEDs^52^. Moreover, tomato seedlings irradiated with red LED or FL exhibited greater leaf area compared to blue or far-red LEDs 15 days after grafting^53^.

The quality and developmental status of grafted watermelon seedlings can be reaffirmed by shoot and root biomass accumulation. In particular, the root system regrowth is a crucial factor and it is important to be achieved as quickly as possible. At 3 days after grafting, root dry weight values were in accordance with the root architecture analysis discussed above, reaffirming the positive effects of B light on the root system development. However, a reversed result is observed after 6 days of light irradiance in the healing chamber. Moreover after 3 days from grafting, two quality indices, R/S ratio and DQI which are calculated from destructive and non-destructive measurements followed the same trend as root dry weight and B light proved beneficial for the two parameters compared to 12B and R (only in R/S ratio). After the second set of lighting (days 4 to 6 after grafting), shoot and root dry weight were inferior under R-B which was also mirrored in DQI values, while B-B seedlings exhibited inferior root dry weight and R/S ratio. These results proved that B light during days 4 to 6 after grafting decelerated biomass accumulation and negatively affected the seedling quality compared to R and 12B. Quite similarly, cucumber seedlings treated with R1/B1 LED enhanced the dry mass compared to narrow-band blue LED^54^, while in another study with grafted watermelon seedlings of the same hybrids, DQI was enhanced under 12B compared to B^55^. Chlorophylls absorb light with peak wavelengths at the red (600–700 nm) and blue (420–450 nm) spectral zones^24^, thus being necessary for photosynthesis and biomass production. As discussed above, seedlings treated with R-B and B-B were among the ones with the least chlorophyll content as well as the lowest PI_ABS_ while they also developed inferior photosynthetic apparatus, which explains the lower biomass production under these treatments.

## Conclusions

Rapid and efficient vascular reconnection between scion and rootstock is critical for the formation of healthy and vigorous grafted seedlings. Watermelon is particularly challenging due to the concurrent removal of the root system which must be developed as quickly as possible. In the healing chamber, B provided the most promising wavelength for the first three crucial days which is depicted by the least damage and faster reparation of the photosynthetic apparatus. Moreover, phytohormones participated in a signaling cascade resulting in the rapid vascular reconnection and subsequently faster root system development under B light. However, after six days seedlings from all light treatments developed sufficient vascular bridges and the picture is reversed. At this time interval, seedlings treated with B for the first 3 days and R for days 4 to 6 after grafting, as well as seedlings treated with 12B at any point had superior development with better photosynthetic characteristics and greater root system development which were reflected in several quality indicators such as leaf area, shoot and root biomass accumulation, and DQI. On the contrary, seedlings treated with R-B performed poorly in almost all qualitative parameters. In addition, B light led to inferior biomass accumulation and seedling quality (R/S ratio and DQI) during days 4 to 6. From the results of this study it is clear that red and blue lights act through phytohormones and play different roles on the vascular reconnection and the subsequent development of grafted watermelon seedlings.

## Supplementary Information

Supplementary Information.
