# Supplementary material for: Blue light promotes vascular reconnection, while red light boosts the physiological response and quality of grafted watermelon seedlings
Source: Sci Rep. 2021 Nov 5;11:21754. doi: 10.1038/s41598-021-01158-w (PMC8571345; doi:10.1038/s41598-021-01158-w)
Supplement: Supplementary file 1 — Supplementary Information. [file 41598_2021_1158_MOESM1_ESM.docx]

**
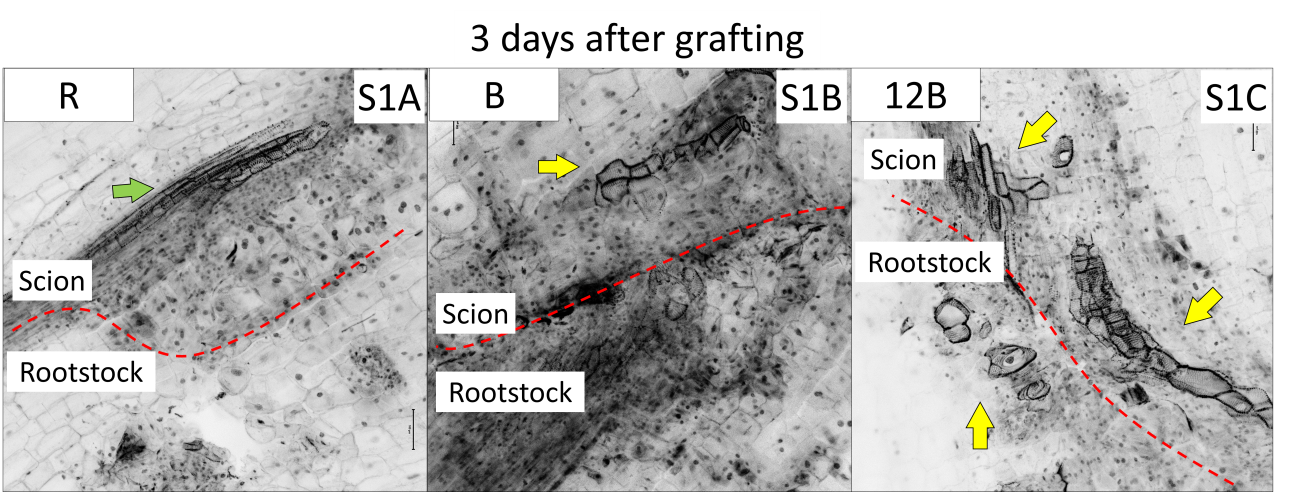
**

**
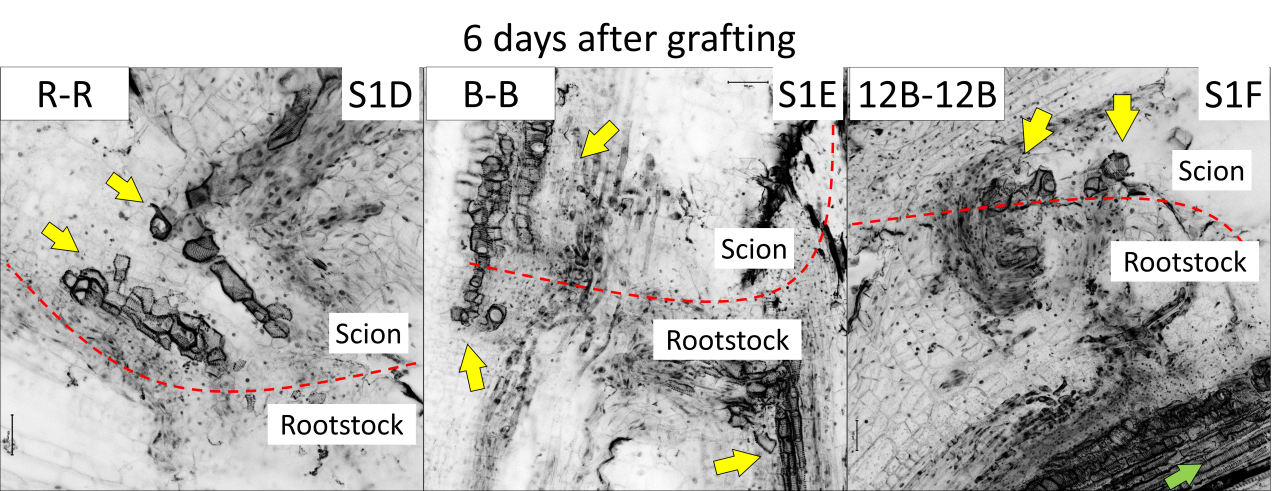
**

**Supplementary Figure S1.** Projections of CLSM sections, depicting the histological organization at the grafting junction of grafted watermelon seedlings after three (upper line) or six (bottom line) days in the healing chamber under three light treatments. In all the CLSM figures, fluorescence intensity has been inverted (negative image) for better visualization. Red dashed lines indicate the grafting junction line. Yellow arrows indicate newly formed vessels which derived from redifferentiated parenchymatic cells. Green arrows indicate initial xylem vessels.

**
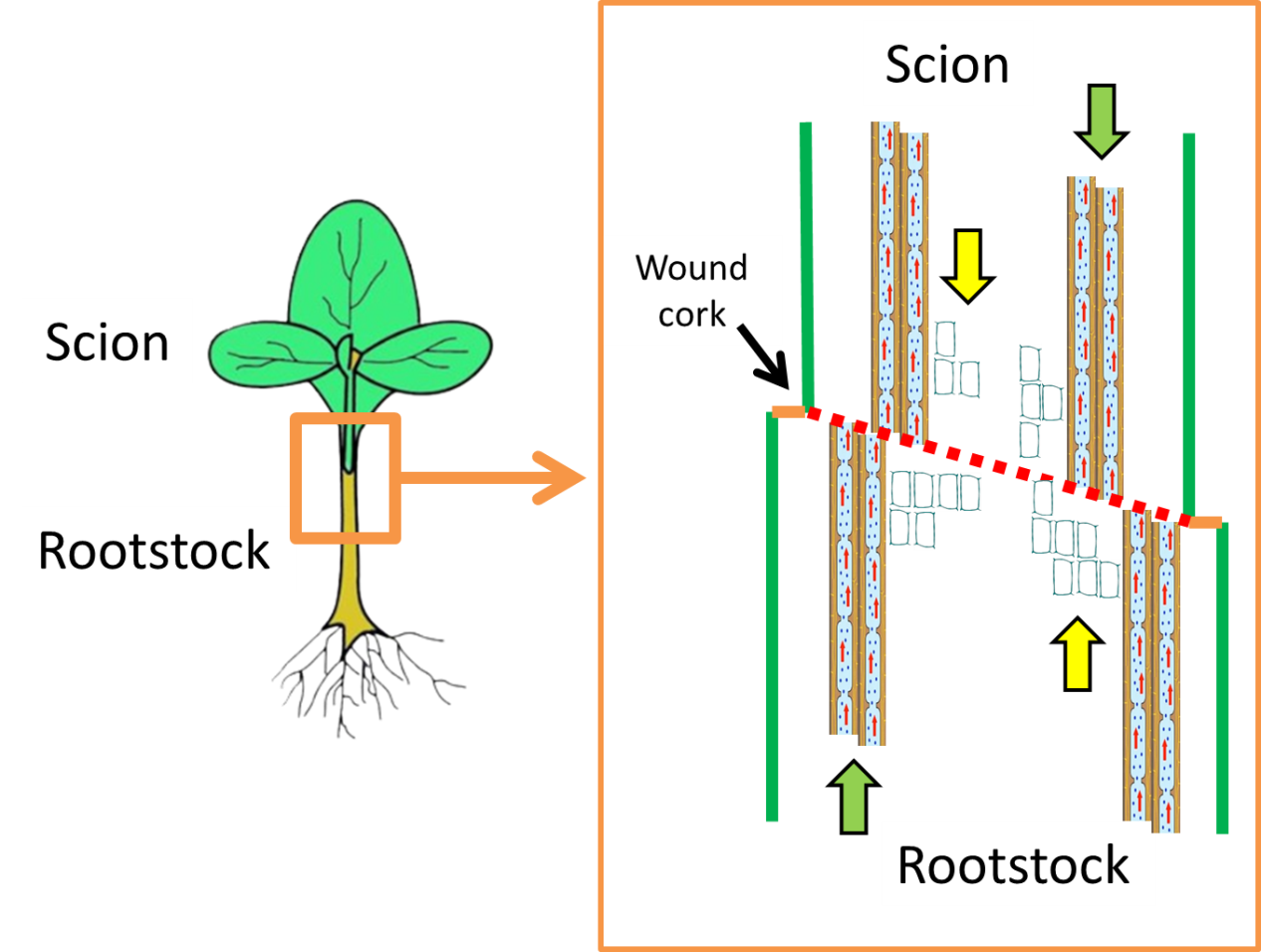
Supplementary Figure S2.** Schematic depiction of histological organization of grafted watermelon seedlings at the grafting junction. The red dashed line indicates the grafting junction line. Yellow arrows indicate newly formed vessels which derived from redifferentiated parenchymatic cells. Green arrows indicate initial xylem vessels.
